# What’s Fair Is Fair: Leveling the Playing Field for Young Scientists

**DOI:** 10.3390/vaccines6020033

**Published:** 2018-05-31

**Authors:** Jonathan W. Yewdell

**Affiliations:** Cellular Biology Section, Laboratory of Viral Diseases, NIAID, Bethesda, MD 20892, USA; jyewdell@niaid.nih.gov

Like every MD Ph.D. pup, I was faced with the BIG decision: medicine or science? It was easy to choose. As I saw it, in the clinic you were expected to do as told and methodically follow a well-trodden career path that tolerated little deviation. In the lab, you were expected to think for yourself and create a career based on your ability to generate new knowledge. Typically, you could start your own group as a principal investigator (PI) after just a few years (sometimes even zero) as a post-doc, and the NIH R01 funding success rate was much higher than it is now (30+% vs. less than 20%). This looked pretty good to my young self. Prizing my independence and placing a high value on creativity, I went straight to the lab. I do not remember having second thoughts or even a backup plan. I don’t think my situation was that unusual. Opportunities abounded for well-trained, ambitious young scientists.

That was in 1981. In the intervening years, many factors have conspired against the independence and success of young scientists.

1. Lengthening of the post-doc period to include the most productive years of a young scientist’s hands-on experimental work (typically mid 30’s). This is clearly reflected in the increased average age for first time R01 recipients from 36 in 1983 to 42 today. This restricts young scientists’ investigational freedom to fit with their post-doc boss’s interests and whims and assigns the lion’s share of the credit for their discoveries to said boss.
2. Extreme, unsustainable, and unhealthy competition in obtaining a PI position, limiting the fraction of young scientists who get the opportunity to blaze their own path, and increasing post-doc career anxiety to debilitating levels.
3. For the fortunate fraction of post-docs who actually land PI jobs, difficulty in obtaining the funding to purchase the saws, axes, worker bees etc. needed for path blazing.
4. Shackles on creativity placed by funding committees that, while charged to support high-risk research, typically insist on just the opposite.
5. The tendency in many fields, including influenza virus research, to assign a significant fraction of total funding to large program grants controlled by one or a few senior scientists. In some cases, publications may have to be cleared by a committee, with the possibility of imposing scientific censorship on findings that threaten established wisdom, particularly that of senior members of the group.
6. The workings of old boy networks in reviewing grants, papers, and selecting conference speakers that inevitably favors established scientists.
7. The increasing influence of luxury journals and their gatekeeping professional editorial staff who in essence decide which research topics are interesting and important. Senior scientists with well-established track records and reputations have a natural advantage of having their papers favorably reviewed, amplified if they are on personal terms with the editors from previous interactions as authors, reviewers, or contacts at meetings or their own institutions when editors go into the field.

Why is it important to level the playing field? Well, let us start with the basic issue of fairness. The Golden Rule of treating others the way you would like to be treated, or more directly, *were actually treated*, certainly applies here. It is hypocritical for senior scientists to stand by idly (or worse) while young scientists struggle up a much steeper path to success. More pragmatically, research is humanity’s golden egg laying goose, essential for improving the standard of living and maintaining economic competitiveness. Young scientists have the energy, focus, and up-to-date skills and knowledge to perform the best experiments themselves, and most effectively supervise and inspire those in the training pipeline. Further, they are far more likely to think originally since they have fewer (or no) findings or theories of their own to defend and are not burdened with a deep knowledge of their field, which, while certainly useful, can cramp creativity. 

By all means, I am not (suicidally) advocating the career decapitation of senior scientists but rather for improving the balance between old and young scientists and the resources at their disposal. The goal is to optimize the knowledge and wisdom of senior scientists with the unbounded energy, enthusiasm, and heresy of the young. 

How to achieve this? There are many elements to reforming biomedical research and I refer readers to my own ruminations and those of others [1,2,3,4,5,6]. Perhaps the most important element is changing the basis for government funding from project-based to people-based grants. This would remove the barriers that grant reviewers impose on creative research. A critical further step would be mandating that institutions recruit young PIs only if they have funding to fully support said young PI during the tenure-track period. This would relieve the enormous burden of fundraising, allowing the young scientists to focus their energy on research, training, and teaching. It will also improve the tenure selection process, which would be based on selecting the best potential scientists and mentors, and not the best fundraisers. Support for tenure-track scientists could come in part from private fundraising but most would likely have to be provided by traditional funding sources (governments and large foundations) who would set aside a portion of their budgets for this express purpose. Resources could be distributed to favor institutions that generate successful young scientists. The NIH intramural program provides a blueprint for such a system, and might even be expanded to include off-site researchers located at universities and research institutes.

Having touted the qualities of young scientists, I urge you to peruse this outstanding issue of Vaccines to see just what young flu vaccinologists are up to.
